# Topical Drug Delivery to the Posterior Segment of the Eye: Addressing the Challenge of Preclinical to Clinical Translation

**DOI:** 10.1007/s11095-018-2519-x

**Published:** 2018-10-29

**Authors:** Gerard A. Rodrigues, David Lutz, Jie Shen, Xiaoda Yuan, Hong Shen, James Cunningham, Hongwen M. Rivers

**Affiliations:** 1 Biological Research, Allergan plc,, Irvine, California 92612 USA; 2 Nonclinical and Translational Sciences, Allergan plc,, Irvine, California 92612 USA; 3 Pharmaceutical Development, Allergan plc,, Irvine, California 92612 USA; 4 Allergan plc,, 2525 Dupont Drive, Irvine, California 92612-1531 USA

**Keywords:** formulation, *in vivo* evaluation, posterior segment, topical delivery, translation

## Abstract

Topical delivery of therapeutics to the posterior segment of the eye remains the “holy grail” of ocular drug delivery. As an example, anti–vascular endothelial growth factor biologics, such as ranibizumab, aflibercept, and bevacizumab, are delivered by intravitreal injection to treat neovascular age-related macular degeneration and, although these drugs have revolutionized treatment of the disease, less invasive alternatives to intravitreal injection are desired. Multiple reports in the literature have demonstrated topical delivery of both small and large molecules to the back of the eye in small animal models. Despite this progress, successful translation to larger species, and ultimately humans, has yet to be demonstrated. Selection of animal models with relevant ocular anatomy and physiology, along with appropriate experimental design, is critical to enable more relevant feasibility assessments and increased probability of successful translation.

## Introduction

Topical instillation of eye drops is non-invasive and the most common route for administering therapeutics to the eye. Although this route is a viable method of drug delivery for the treatment of anterior segment diseases, it remains a major challenge to efficiently deliver drugs topically to treat posterior segment diseases such as age-related macular degeneration (AMD) and diabetic macular edema. Static and dynamic barriers limit penetration of therapeutic molecules into the ocular tissues (1,2). Static barriers to drug transport include the corneal epithelium, conjunctival epithelium, sclera, choroid, Bruch’s membrane, and retinal pigmented epithelium; these work together with dynamic barriers such as choroidal and conjunctival blood flow, lacrimation, and lymphatic drainage and efflux to efficiently reject foreign substances and pathogens. As a result, bioavailability is low following topical dosing of eye drops, with typically less than 3% of topically administered drug reaching the aqueous humor (1) and even less reaching the posterior segment, resulting in subtherapeutic drug concentrations in these tissues (3).

Despite the large unmet need and market opportunity, topical delivery of hydrophilic macromolecule drugs such as therapeutic proteins to the posterior segment remains particularly challenging. The current standard of care for the treatment of AMD is intravitreally administered anti–vascular endothelial growth factor (anti-VEGF) biologics. A number of registration trials have established the recommended frequency of these injections, which is typically monthly or bimonthly depending on the drug. In addition to the significant treatment burden for patients and caregivers, frequent intravitreal injections increase the risk of complications including endophthalmitis, cataracts, retinal detachment, and vitreous hemorrhage. In real-world experience, patients may receive fewer treatments than those participating in clinical trials and, as a consequence, have poorer-than-expected treatment outcomes (4). Clearly, achieving effective delivery with a less invasive route of administration could provide significant benefit to patients. Representative studies that have investigated topical administration of molecules in preclinical models are listed in Table I. This article discusses the key considerations in evaluating topical drug delivery for treating retinal diseases, with an emphasis on translation from preclinical models to humans.

**Table I:** Examples of Topically Administered Molecules Investigated in Preclinical Models

Compound	Formulation	Preclinical data	Reference
TG100801	Solution	CNV model in mouse, edema in rat	(5)
Pazopanib	Solution	CNV model in rat	(6 7)
Acrizanib	Suspension	CNV model rat and mouse	(8)
Memantine	Solution	Drug levels in retina of rabbit	(1)
Dorzoamide	Solution	Drug levels and carbonic anhydrase activity in corneal endothelial cells, ciliary body, lens epithelial cells, and retina in rabbit	(13)
Dexamethasone	Iontophoresis	Drug levels in retina and vitreous of rabbit	(14)
Bevacizumab	Solution	Drug levels in iris/ciliary body, vitreous, retina/choroid, and plasma in rabbit	(16)
Anti–intercellular adhesion molecule-1 antibody	Solution by osmotic pump	Drug levels and VEGF-induced leukostasis in the choroid and retina in rabbit	(17)
28-kD single-chain antibody fragment	Sodium caprate	Drug levels in vitreous in rabbit	(18)
Bevacizumab	Annexin A5–based liposomes	Drug levels in retina of rat and rabbit	(19)
Transforming growth factor beta 1	Annexin A5–based liposomes	Drug levels in vitreous in rabbit	(20)
Acidic fibroblast growth factor	CPP (TAT)	Ischemia reperfusion model in rat	(22)
Calpain inhibitory peptide	CPP (TAT)	Drug levels in retina of rabbit	(23)
Green fluorescent protein	CPP (POD)	Drug levels in cornea of mouse	(24)
Bevacizumab	CPP (R6)	Drug levels in vitreous and retina in rat and CNV model in mouse	(25)

### Preclinical Evaluation: Animal Models and Translatability

Many preclinical studies claiming successful topical delivery of small molecules or proteins to the retina have used rodent models (Table I). Advantages include the availability of established and well accepted pharmacological models, and practical considerations such as cost and availability. Despite these advantages, the rodent eye is anatomically dissimilar to the human eye, and resulting differences in the distribution and elimination of drugs can have a significant impact on the pharmacodynamic response. Thus, efficacy data generated using rodent models should be interpreted with caution.

The importance of choosing the appropriate preclinical animal model is underscored by multiple failures of topically administered compounds in clinical trials, despite promising preclinical data in rodents. For example, TG100801, a small-molecule multikinase inhibitor, was shown to suppress laser-induced choroidal neovascularization (CNV) in a mouse model, as well as edema in a rat model of retinal vein occlusion, following topical administration (5). TG100801 is a prodrug selected for its ability to penetrate the cornea; the active inhibitor is then generated by hydrolysis (5). However, no pharmacodynamic studies were conducted in a larger species, and when tested in humans, although well tolerated, TG100801 failed to demonstrate efficacy in AMD patients (ClinicalTrials.gov identifier: NCT00509548). Similarly, pazopanib, a small-molecule multikinase inhibitor formulated as an eye drop at 5 mg/ml and dosed topically twice daily, significantly decreased leakage in a laser-induced CNV model in rats by ~90% (6). However, when it was later tested as a 10-mg/ml eye drop dosed four times daily for 12 weeks in patients with subfoveal CNV secondary to AMD, it did not decrease central retinal thickness (7). A very similar outcome was recently reported for acrizanib, a VEGF receptor-2 (VEGFR-2) inhibitor specifically designed for topical ocular delivery to treat neovascular AMD (8). The rodent CNV model (both mouse and rat) was used as the primary means of developing structure-activity relationships and screening VEGFR-2 inhibitors with potent *in vitro* activities for topical delivery. Acrizanib showed a 99% inhibitory effect following three times daily dosing as a 1% suspension in the mouse CNV model, whereas 94% inhibition was achieved when dosed as a 3% suspension twice a day in the rat CNV model. Despite the positive data in both rodent models, acrizanib did not demonstrate efficacy in a proof of concept study in patients with neovascular AMD (ClinicalTrials.gov identifier: NCT02355028). In spite of these failures, topical delivery of multikinase inhibitors to the posterior segment continues to be an active area of research. For example, a topical suspension formulation of a multikinase inhibitor, PAN-90806, is currently being tested in AMD patients in a phase I/II trial (ClinicalTrials.gov identifier: NCT03479372).

**Fig. 1:**
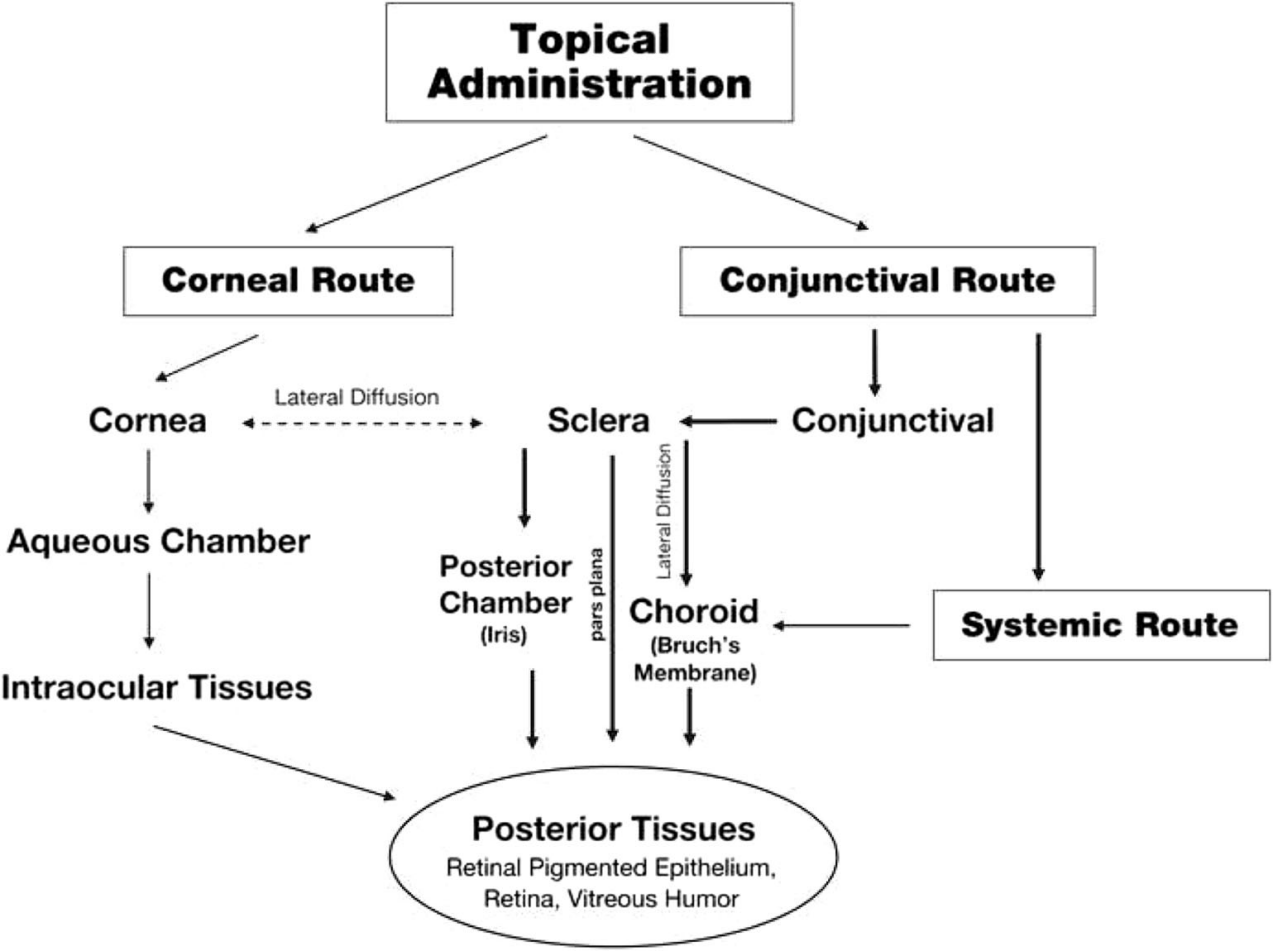
Drug distribution pathways through the corneal and conjunctival/scleral routes following topical administration. Reprinted from Advanced Drug Delivery Review, Vol 57, Hughes PM, Olejnik O, Chang-Lin JE, Wilson CG, Topical and systemic drug delivery to the posterior segments, Pages 2010–32, Copyright 2005, with permission from Elsevier.

Compared with rodents, the relevant anatomical and physiological parameters in rabbits are more similar to those in humans (9). Rabbits share ocular features with humans including a comparable size, vitreal volume, and internal structure, and thus a similar diffusional path for topically administered compounds to reach the posterior segment (9). Additionally, the intravitreal pharmacokinetic parameters in rabbits have shown predictable correlations with those in humans (10). The rabbit is also relatively easy to handle and is the most economical of the larger species models. Importantly, an increasing number of rabbit models of ocular diseases, including AMD, have been established (9). Even preclinical data generated through use of a larger animal species such as rabbits should be interpreted with caution due to anatomical/physiological differences that have the potential to impact drug disposition. For example, rabbits have a lower blinking rate than humans, which would be expected to increase the residence time of topically administered drug formulations and potentially affect the bioavailability of drugs in intraocular tissues (11). In addition, the proportionally larger anterior segment and more viscous vitreous humor in rabbit relative to human eyes may also affect distribution of drugs that rely more predominantly on a corneal route of diffusion (Fig. 1). Finally, animals with healthy eyes are typically used in pharmacokinetic studies. This may result in underestimation of drug clearance when extrapolated to diseased human eyes with a compromised blood-retinal barrier (12).

In addition to selection of an appropriate animal model, proper study design is critical to avoid incorrect interpretation of study results with regard to drug distribution. Topically administered drugs may enter the eye via the cornea and/or the conjunctival epithelium. Penetration of drugs to posterior segment tissues following corneal absorption involves transit through the anterior segment before reaching the vitreous and the retina. Following absorption into the conjunctiva, drugs can either diffuse through the sclera or the cornea, or alternatively, be cleared into the systemic circulation before reaching the vitreous and retina (1) (Fig. 1). In our experience, assessment of drug levels and/or efficacy in the contralateral, untreated eye is critical to eliminate a potential confounding factor of drug distribution from the systemic circulation following topical administration. Given the much larger blood volume in humans *vs*. rabbits (~5 l in an adult human *vs*. ~0.12 l in a 2-kg adult rabbit), drug distribution to the back of the eye via systemic circulation following topical administration in rabbits may result in tissue levels that do not translate to humans.

To date, the reported preclinical successes of posterior segment drug exposure from topical dosing in a rabbit eye have been primarily observed with small molecules (3) (Table I). For example, topical instillation of 35 μl of 0.1% memantine aqueous solution twice daily for 7 days was able to achieve drug levels in the retinal tissue sufficient for retinal neuroprotection in a rabbit model (1). In another example, retinal concentrations of dorzolamide adequate for inhibition of carbonic anhydrase isoenzyme II in rabbits were achieved using a once-daily topical dose of 1% aqueous dorzolamide HCl solution (13). Iontophoresis, the use of electrical current to enhance tissue penetration, is a topical drug delivery approach that has shown some promise. Although the number of studies using transcorneal iontophoresis for posterior segment delivery is limited, there are multiple examples of successful transscleral iontophoresis of small molecules, including antibiotics and steroids, into the vitreous of rabbits (14). Iontophoretic dexamethasone was advanced into a phase Ib/IIa clinical trial (ClinicalTrials.gov identifier: NCT02485249) for patients with macular edema before the trial was terminated due to enrollment issues.

While there has been some success in the topical delivery of small molecules to the posterior segment in a large eye, the hurdles for biologics are higher given the much larger molecular weight and polarity. It has been shown that scleral permeability decreases with increasing molecular weight and molecular radius (15). On the other hand, biologics typically have higher affinities for their target than small molecules. Thus, the challenges associated with lower corneal or scleral penetration may be offset to a certain degree by a lower drug concentration needed at the target to achieve efficacy. However, even the potent biologic bevacizumab failed to achieve therapeutic concentrations in the retina and vitreous of rabbits following aggressive topical dosing of 1.25 mg/0.05 ml six times daily for a week (16). Ambati *et al.* (17) reported successful transscleral delivery of a monoclonal antibody directed against intercellular adhesion molecule 1 to the retina and choroid in rabbits without detectable systemic exposure, resulting in inhibition of VEGF-induced leukostasis. However, this required use of an osmotic pump delivering drug constantly over a 24-h period to the superotemporal scleral surface (17).

To address the challenge of topical protein delivery, different formulation approaches have been explored to attempt to overcome barriers and reach therapeutically meaningful drug levels in the posterior segment (Table I). Williams *et al.* (18) reported detection of a 28-kDa single-chain variable domain antibody fragment in the rabbit vitreous at concentrations of 50–150 ng/ml 12 h after topical dosing of the antibody fragment with a permeability enhancer, sodium caprate. Interestingly, penetration of its corresponding full-length (IgG) antibody into the vitreous was not observed (18). Davis *et al.* (19) reported delivery of physiologically relevant concentrations of bevacizumab to the back of the eye in both rats and rabbits using annexin A5–associated liposomes. Similarly, Platania *et al.* (20) reported the use of an annexin A5–based liposome formulation to deliver high levels of transforming growth factor beta 1 to the rabbit vitreous following topical administration. The use of cell-penetrating peptides (CPPs) is another formulation approach that has been investigated for ocular delivery of proteins and peptides (21). However, preclinical studies to date using this approach have been largely limited to rodent models. For example, Wang *et al.* (22) used the CPP HIV transactivator of transcription (TAT) to deliver acidic fibroblast growth factor to the rat retina following topical administration at levels sufficient to protect the retina from ischemia–reperfusion injury. Similarly, Ozaki *et al.* (23) demonstrated delivery of topically administered calpain inhibitory peptide conjugated to TAT to the posterior segment of the rat eye. Another relevant example is the use of a CPP, peptide for ocular delivery (POD), conjugated to green fluorescent protein to enable its uptake by corneal epithelium when the formulation was topically administered to mouse eyes (24). Most recently, de Cogan *et al.* (25) reported that therapeutic levels of bevacizumab in the posterior segment were achieved in rats following topical administration when the antibody was combined with a CPP, poly-arginine-6. Notwithstanding these encouraging results in rodents, the translational application of these platforms is still in its infancy. Moreover, the enhancement of corneal permeability through the use of agents such as sodium caprate has the potential to cause ocular toxicity associated with compromised interepithelial tight junctions (3). Therefore, in addition to the evaluation of efficacy, thorough safety assessment in a non-rodent species is warranted for these novel formulation-based methods of topical delivery (26).

## Conclusions

Although topical ocular drug administration represents a less invasive and therefore safer route of dosing than intraocular injections, clinical success in delivering drugs to the posterior segment by this route remains elusive. There are now multiple examples of preclinical pharmacokinetic and efficacy data generated in rodent models failing to translate to humans. The absence of translation from rodents to humans is not surprising when anatomical and fluid dynamic differences in the eye across species are considered. Based on parameters including, but not limited to, corneal thickness, aqueous humor volume and flow rate, vitreal volume, and circumferential or linear distance from the ocular surface to the back of the eye, one would expect delivery of drugs to the posterior segment via topical dosing to be more difficult in humans than in rodents. Unfortunately, rodents remain the species of choice for many feasibility studies evaluating topical delivery. We advocate for the use of larger species such as rabbits, dogs, pigs, or monkeys when testing novel drug delivery approaches to maximize the probability of predictive preclinical assessment and successful clinical translation. In addition, assessment of ocular pharmacokinetics in both the treated and contralateral eyes as well as in plasma is important to inform the delivery mechanism. Lastly, thorough safety assessment, which can and should be considered as part of the study design, is also critical. With the continued innovation of novel delivery approaches, we remain hopeful that topical delivery of drugs, including biologics, to the posterior segment of the eye may become a reality in the future to address a significant unmet medical need.

## Abbreviations

AMD: Age-related macular degeneration
CPP: Cell-penetrating peptide
CNV: Choroidal neovascularization
TAT: Human immunodeficiency virus (HIV) transactivator of transcription
POD: Peptide for ocular delivery
VEGF: Vascular endothelial growth factor
VEGFR-2: VEGF receptor-2
